# The Implementation of Mindfulness-Based Cognitive Therapy: Learning From the UK Health Service Experience

**DOI:** 10.1007/s12671-012-0121-6

**Published:** 2012-06-22

**Authors:** Rebecca S. Crane, Willem Kuyken

**Affiliations:** 1 Centre for Mindfulness Research and Practice, School of Psychology, Bangor University, Gwynedd, LL57 1UT UK; 2 Mood Disorders Centre, School of Psychology, University of Exeter, Exeter, EX4 4QG UK

**Keywords:** Mindfulness-based cognitive therapy, Knowledge transfer, Implementation, Effectiveness, Health service

## Abstract

Mindfulness-based cognitive therapy (MBCT) is an effective depression prevention programme for people with a history of recurrent depression. In the UK, the National Institute for Clinical Excellence (NICE) has suggested that MBCT is a priority for implementation. This paper explores the exchange, synthesis and application of evidence and guidance on MBCT between the academic settings generating the evidence and delivering practitioner training and the practice settings where implementation takes place. Fifty-seven participants in a workshop on MBCT implementation in the NHS were asked for their experience of facilitators and obstacles to implementation, and a UK-wide online survey of 103 MBCT teachers and stakeholders was conducted. While MBCT is starting to become available in the NHS, this is rarely part of a strategic, coherent or appropriately resourced approach. A series of structural, political cultural, educational, emotional and physical/technological obstacles and facilitators to implementation were identified. Nearly a decade since NICE first recommended MBCT, only a small number of mental health services in the UK have systematically implemented the guidance. Guiding principles for implementation are set out. We offer an implementation resource to facilitate the transfer of MBCT knowledge into action.

## Introduction

Even if a psychosocial intervention has compelling aims, has been shown to work, has a clear theory-driven mechanism of action, is cost-effective and is recommended by a government advisory body, its value is determined by how widely available it is in the health service. Frameworks for treatment development see implementation as the final challenge for any new intervention (Medical Research Council 2008). Mindfulness-based cognitive therapy (MBCT) is a psychosocial group-based relapse prevention programme developed from translational research into mechanisms of depressive relapse/recurrence (Segal et al. 2002). MBCT is effective compared with usual care and maintenance antidepressants (Piet and Hougaard 2011), probably cost-effective (Kuyken et al. 2008), has evidence of mechanism (Kuyken et al. 2010), has published standards for teachers (Crane et al. 2010) and has been recommended by the National Institute for Clinical Excellence (NICE) (2009) as a psychological approach in preventing depressive relapse among patients with three prior episodes of depression. There is much enthusiasm among health professionals for training in MBCT as evidenced by the establishment of MBCT teacher training programmes in many countries including the UK, the Netherlands and Australia.

Depression is a major public health problem that tends to run a relapsing and recurrent course (Judd 1997), producing substantial decrements in health and wellbeing (Moussavi et al. 2007). Currently, the majority of depression is treated in primary care, and maintenance antidepressant treatment is the mainstay approach in preventing relapse/recurrence (Geddes et al. 2003; Katon and Schulberg 1992). Where psychosocial treatments are available, they tend to be focussed on the treatment of current depression using cognitive behavioural therapy (Pilling et al. 2011); there is rarely a focus on psychosocial prevention programmes such as MBCT.

The investment and infrastructure for translating evidence into practice is generally more ad hoc than that for producing and synthesising the evidence. Implementation relies on achieving significant and planned whole system change involving individuals, teams and organisations (Rycroft-Malone 2010). Evidence has shown that the processes involved in transferring research to practice settings are complex, interconnected and multifaceted (Damschroder et al. 2009). A recent study suggests that organisations that were successful in overcoming barriers to implementation shared two common factors: (1) they had adapted generic quality improvement strategies to fit their context and circumstances and (2) they had simultaneously addressed challenges inherent in quality improvement in structural, political, cultural, educational, emotional and physical/technological areas (Bate et al. 2008).

This paper considers the current status of MBCT implementation and the facilitators and barriers to implementation, using the UK health service as an exemplar. It aims to (1) investigate how far MBCT has been implemented to date, (2) review the barriers and facilitators to implementation and (3) make recommendations that policy makers, service managers, MBCT teachers and patient groups can use to develop MBCT services.

## Method

Our aims were met through capturing themes expressed by participants in a workshop on MBCT implementation and through undertaking a survey of MBCT teachers and stakeholders. The research had ethical approval from the Bangor University School of Psychology Ethics Committee. We also present four anonymised exemplars of implementation of MBCT in practice.

### The Implementation Workshop

The implementation workshop was part of the Mindfulness Now conference at Bangor University in 2011 and was led by the authors of this paper. Fifty-seven participants attended. All had chosen the workshop because they had an interest in MBCT implementation, either from the perspective of being an MBCT teacher or because they were strategically involved in MBCT service development. Participants worked in small groups to share experiences on these three questions: (1) what is the state of implementation within your organisation, (2) what has proved most challenging while developing MBCT services in your organisation and (3) what factors have proved most important in supporting the development of MBCT in your organisation? Key themes were identified from recordings of small group discussions and the flip chart notes generated by the small groups.

### The Survey of Experiences of Implementation

The survey of experiences of implementation was developed to assess the national status of MBCT services in the NHS exploring (1) the extent to which MBCT services are being implemented in line with national guidance, (2) respondents' perceptions of the barriers and facilitators to MBCT implementation in their organisation, (3) how the national guidance is being interpreted during implementation and (4) practical issues which influence implementation. The survey was initially conducted by post and subsequently developed as an online survey to increase uptake. A purposive sampling process was employed to enable us to gather information from people who clearly know what MBCT is and who can report on the status of implementation in their organisation. We circulated the questionnaire to the databases of the Bangor, Exeter, Oxford and Scottish mindfulness-based training organisations reasoning that this would select people who were involved in mindfulness work and therefore able to report on the status of implementation in their organisation. The majority of the 103 respondents were MBCT teachers working in the NHS, involved in delivering or developing MBCT services. All the broad geographical areas in the UK were represented, although there was a higher response rate from several regions (e.g. the South West of England 26 %, Scotland 14 % and London 14 %) and comparatively a low response rate from other regions (e.g. Yorkshire and the Humber and Northern Ireland 1 % each).

### Exemplars

Consent was gained to gather the experiences of MBCT service leads in four geographical areas of the UK.

## Results

The following section reports the results of the survey and the themes that emerged during the workshop discussions. Each small group within the workshop was given two of the six challenges inherent in quality improvement (Bate et al. 2008) as a focus for their discussion. Themes were extracted by listening to recordings of these discussions and were captured under the “challenges” headings.

### The Current Status of Implementation in the NHS

#### The Extent to Which MBCT Services are Being Implemented

While the study design does not enable us to make definitive inferences about the take-up of MBCT across the UK, the results are indicative of considerable variability in availability of MBCT. Although only 18 % of respondents reported that their organisation had a strategic plan that included MBCT, 9 % reported a thriving MBCT service and 32 % had “services that are slowly improving but there is a long way to go”, indicating that some MBCT services are developing in the absence of organisational strategy; 32 % reported that the development of MBCT services is well supported by clinical staff but progress is slow because of limited resources. Over half (59 %) either had no service or a service that does not play a major role and operates with little organisational support. To explore whether having an organisational strategy was associated with indicators of successful MBCT implementation, we computed simple non-parametric Spearman two-tailed correlations. Where respondents indicated that their organisation had a strategic plan to implement MBCT services, they were also more likely to indicate that MBCT was widely available; therapists were supported in terms of training, supervision and dedicated time; referrals were sufficient and appropriate; referrers had a good understanding of MBCT and MBCT classes were resourced in terms of physical space and administrative support (all Spearman rhos significant at *p* < 0.05).

#### How the NICE Depression Guidance on MBCT is Being Interpreted

The group of patients for which MBCT has the most robust evidence base are those vulnerable to recurrent depression and currently in remission. In line with NICE guidance (National Institute for Clinical Excellence 2009) and SIGN Guidelines in Scotland (Scottish Intercollegiate Guidelines Network 2010), 78 % of respondents reported that this was the patient group for whom classes were offered. In addition, 60 % of respondents reported that MBCT is also being offered to patients with recurrent anxiety, and 47 % reported that it is offered to other client groups (e.g. chronic pain/fatigue). Several respondents expressed a hope that the evidence base would expand to a wider group. Interpreting the emergent evidence base was expressed as a barrier to implementation.

## Implementation Barriers and Facilitators

Respondents' views are presented using the six core challenges in quality improvement (structural, political, cultural, educational, emotional and physical/technological) as a framework (Bate et al. 2008).

### The Structural Challenge

Structuring Quality Improvement Efforts and Embedding Them Within the Organisational Fabric

Several workshop participants reported working to implement MBCT with little strategic support, with the provision of MBCT sometimes relying on a single clinician. Half of respondents reported that there was no managerial support to prepare or deliver classes within clinical time. Only 35 % reported that delivering MBCT classes was explicitly within their role. Many described how the UK Good Practice Guidelines for Mindfulness-based Teachers have been supportive in framing local governance criteria for running MBCT classes and facilitating communication to service managers (UK Network 2011).

The survey results indicated that MBCT services are currently developing equally in primary and secondary care. In some organisations, the focus on management of acute presentation rather than prevention presented a barrier to implementation. In other organisations, the NICE (2009) recommendations had given MBCT a natural place in service developments.

### The Political Challenge

Negotiating the Politics of Change and Securing Agreement on Common Goals

In the UK, MBCT implementation is taking place within the current context of politically initiated restructuring and strong budgetary constraints. Workshop participants shared experiences of how challenging it was to develop high quality MBCT services alongside strong organisational agendas that pressured a focus on output, performance and outcomes, emphasising “quick wins”. In the words of one workshop participant: “It is difficult to hold ground with something that is steady, measured, and with the longer term in mind when surrounded by a quick culture.”

On the other hand, outcome evaluation has become routine, and 79 % of survey respondents indicated that emerging MBCT services collect outcome data. This was cited as an implementation facilitator because it enabled services to demonstrate MBCT's acceptability and efficacy in line with the emphasis on outcome-based commissioning.

### The Cultural Challenge

Building Shared Understanding and Commitment to Quality Improvement

The upsurge in interest in mindfulness presents both a challenge and facilitator to the implementation process. On the plus side, service managers tend to be increasingly aware of mindfulness having potential for their organisation. However, respondents described that they frequently felt a lack of understanding from the organisation about what is involved in delivering MBCT classes and a tendency to perceive mindfulness as a panacea.

Workshop participants underlined that MBCT requires a strong personal commitment both to a mindfulness meditation practice and an in-depth training and development process. Some of the workshop participants' colleagues who are not involved in delivering MBCT find mindfulness-based interventions difficult to understand, and participants reported that this tended to result in their colleagues seeing MBCT as a lower priority for development. These tensions can create a cultural barrier to MBCT implementation. One workshop participant summarised her organisation's attitude as “it [mindfulness] is all very well but a bit of a luxury in these hard times.”

Conversely, mindfulness training appeals to health care staff because it can support them in “living well” both at home and at work. Several respondents reported that mindfulness training is offered as part of their organisation's strategy for staff wellbeing. This has positive effects on a number of levels, including developing a culture and “critical mass” of mindfulness within the organisation, which supports the development of clinical services. Mindfulness training for staff was seen as enabling them to “be a steady rock amidst so much change and challenge”.

Workshop participants spoke of the importance of challenging the prevailing culture that puts priority on addressing the “problem” of the acute presentation of anxiety and depression but does not invest in the development of long-term resilience.

### The Educational Challenge

Developing Formal and Informal Learning

The survey indicated a chasm between published standards for MBCT training (Crane et al. 2010) and the reality of what training had been undertaken by practitioners offering MBCT: as many as 36 % reported delivering MBCT without any formal training, and a modal response was that teachers had participated in mindfulness teacher training retreats but had not undertaken a formal postgraduate training in MBCT (54 %). A significant number (17 %) indicated that their organisation saw professional training in a core profession as sufficient to teach MBCT, even though these trainings do not include MBCT within their curricula. The disparity between the widely recognised training standards and reality is probably because the financial support for training clinicians to deliver MBCT is low, with 67 % reporting no support and 66 % reporting no ongoing support to maintain good practice through attending continuing education or supervision.

When asked which professionals teach MBCT within their organisation, psychologists were the largest group (83 %), with occupational therapists (58 %), social workers (44 %) and psychiatric nurses (55 %) also delivering classes. Other professionals included CBT therapist, dietician, family therapist, psychiatrist and physiotherapist.

A theme in the workshop was the mismatch between the duration of time that it takes to cultivate MBCT teaching skills and the cultural tendency within the NHS to “get people trained quickly, get them delivering and then get them training and supervising others”. There was a strong theme in both the survey and workshop that mindfulness practice and teaching needs to be seen as a long-term investment.

Another aspect of the educational challenge is the level of knowledge that referrers have of MBCT; 60 % reported that referrers do not have a clear understanding of the intentions of MBCT and half reported that it is difficult to get sufficient and appropriate referrals.

### The Emotional Challenge

Inspiring and Motivating Staff to Join and Sustain the Improvement Effort

A consistent theme from respondents and workshop participants was the importance of having a “champion” within their organisation who steers the process of change. Ideally, the champion(s) has training in MBCT and has strategic influence within the organisation.

### The Physical and Technological Challenge

Developing a Physical and Technological Infrastructure that Enables Service Improvement

As a group-based intervention that involves teaching a range of mindfulness practices, there are a number of physical and technical requirements to be able to run MBCT classes well; 62 % of respondents reported that there is no fit for purpose room within their organisation in which to deliver classes, and 72 % reported a lack of administrative support for setting up and running MBCT classes. Workshop participants reported that clinicians in their organisation did all the photocopying, room booking and appointment scheduling for setting up MBCT classes.

## Exemplars of Implementation in Practice

Whilst preparing for the workshop, to enable us to investigate the influences of implementation barriers and facilitators in local contexts, we invited MBCT clinicians in various geographical areas within the UK to share their particular experiences of MBCT implementation. We asked four questions: (1) Is there a local strategy for MBCT implementation and if so what are its aims? (2) What is the current status of implementation in your organisation? (3) What factors have been important in facilitating implementation? (4) What factors have been obstacles to implementation? We present four anonymised exemplars of MBCT implementation in practice to illustrate particular factors which facilitate or impede implementation (Table 1). Exemplar 1 illustrates how even in the presence of strong grassroots interest and proximity to an MBCT training programme the provision of MBCT services is inconsistent when there is no organisational strategy or leadership overseeing the implementation. Exemplar 2 illustrates a collaborative implementation process led by a university and local health trust which illustrates how a strong working relationship between a university and primary care trust can provide the elements for a successful MBCT service. However, in the early stages, this relied heavily on a local champion and sustainability was only achieved once a critical mass of referrers, MBCT therapists and patient advocates had built up. Exemplar 3 is an example of a successful MBCT implementation in an NHS trust. It illustrates how strategic leadership within the organisation from individuals who have training in the approach, provision of funding for staff training and grassroots interest from staff who want to train in and implement MBCT all contribute towards the development of a sustainable, well-governed service. Exemplar 4 is an example of successful implementation across an entire region within the UK. It illustrates that the transfer of research to practice thrives when the initiative is given regional strategic support, leadership and funding which enables the development of centralised governance on practitioner training and good practice. Table 1 offers a summary of each of these implementation examples.

**Table 1:** Summary of four examplars of MBCT implementation in the UK

Area	Summary of development	Implementation barriers	Implementation facilitators
Exemplar 1	Classes intermittently available in community mental health teams since 1999	Lack of strategic direction and leadership	Proximity to a mindfulness-based training and research centre
		MBCT service depends on enthusiasm of particular clinicians and therefore ceases when they move post	
		Health board reorganisation leading to strategic uncertainty	Grassroots enthusiasm from clinicians who want to offer MBCT classes to their clients and who are willing to give their personal time to ensure they happen
	Inclusion criteria broadened to include anxiety and depression-related disorders	Funding constraints which limit the provision of training	
	About 24 classes delivered providing a service to 276 clients	Clinicians who are trained in MBCT were not given protected time to deliver classes	Staff willingness to self-fund their training
	One pilot class delivered and researched in primary care setting in 2010—research underlined its effectiveness for patients and that referrers valued the provision of the service for their patients	Organisational preference for individual rather than group interventions	

Exemplar 2	Started as a solo therapist offering MBCT classes (2003–2005) and then offered by two teachers as part of an MBCT research trial (2005–2007), which led to MBCT being commissioned as part of primary care mental health provision (2007–date)	No strategic plan that included MBCT nor careful consideration of depression care pathway	Over time (5–10 years) becoming known regionally for providing MBCT (with GPs and other services)
			Research context provided resource for training teachers and setting up services
			Excellent relationship with NHS primary care mental health commissioners
			Staying close to the MBCT treatment manual and ensuring referrals and patients were appropriate
			Routine reporting of acceptability and outcome data
	The university established a training programme in 2008 which has led to a larger pool of MBCT teachers who are now offering MBCT/MBSR in a range of settings across the region, including MBSR staff groups, mindfulness groups for carers and mindfulness groups for people with chronic physical health problems	MBCT teachers in the NHS unable to secure funding for training	Care over selecting committed, competent teachers
			Care over selecting committed, competent teachers
			Supporting teachers in their learning
			Former MBCT participants have acted as advocates of the service with GPs, the local media and commissioners
			Ongoing reunions 4 times a year for all former MBCT participants is highly motivating both for participants and teachers

Exemplar 3	Part of trust wide strategy to increase access to psychological therapies	Limited resources—demand for the classes is greater than can be provided for by currently trained staff; lack of money for CDs, books and photocopying and printing	MBCT being in the NICE guidelines
			Strategic trust-wide approach with clear management structures
		Practical constraints (e.g. limited availability of teaching rooms)	Trust-wide strategy group consists of clinicians who are trained in MBCT and therefore understand the requirements of the teaching process
	Development of critical mass needed to deliver MBCT to patient groups integrated with provision of mindfulness training for staff for personal and professional development	Competing clinical and management demands of individuals who lead the strategic direction of the implementation process	Strategically influential staff have trained as MBCT teachers and act as champions to facilitate the implementation process
	Since 2008, 12 client groups serving 105 patients and 8 staff groups serving 65 staff have been delivered	The challenge of scheduling the MBCT 8-week course within the constraints of other service demands and staff holidays	Provision of staff taster, professional development workshops and conferences on MBCT
	Collaborating with Bangor and Oxford Universities to develop staff MBCT teaching expertise	Debates with psychiatry over the meaning of recurrent depression	Positive feedback from patients and staff who have taken the class
	Target patient group is those vulnerable to recurrent depression		Increasing media interest in mindfulness
	MBCT special interest group for staff is in place		Outcome data being collected on patient classes

Exemplar 4	Government run body has developed a strategy for implementing evidenced-based psychological therapies across the region. MBCT is part of this because of its recommendation by NICE	Occasional lack of support from local management which is usually easily addressed because of the presence of a central strategy	Central strategic lead enabling a regional focus for development
	Systematic process of building capacity in MBCT teaching skills has been in development since 2005	Recent budgetary constraints in the NHS have reduced time that trained MBCT teachers can dedicate to the teaching development	Trained MBCT/MBSR teachers had strategic influence within government and so championed the development of central initiatives
	There are now NHS clinicians trained to deliver MBCT within most areas in region	Budgetary constraints for staff training process	Two implementation leads are paid centrally to take a strategic view of the process of training, supervision and implementation
	Referral criteria for classes have been broadened to include anxiety and depression-related disorders		A regional forum of mindfulness leads from each locality is in place
	Work has taken place on specifying standards for both teachers and supervisors		Supervision courses for experienced mindfulness teachers are being delivered to ensure that good practice provision of supervision for teachers can be met
			Evaluations of all mindfulness activity (patient MBCT classes and staff teacher and supervisor training) are routinely collected

## Discussion

The sample of participants in the workshop and survey was likely to be proactive and strongly involved in promoting MBCT which may have influenced the results. However, taken together, the results of the survey and the workshop themes reveal that MBCT is being inconsistently implemented across the UK, with a small proportion of respondents reporting thriving MBCT services (9 %) alongside the remainder reporting that the implementation of MBCT has either not yet begun in their organisation, has little organisational support or is struggling to manage to develop with the available resources. It is clear that service provision on the ground falls well short of that envisaged in the UK national guidance for MBCT implementation (NICE 2009) and many other international evidence syntheses (e.g. Fjorback et al. 2011).

Many comments made by respondents suggest that provision of classes relies heavily on the commitment and dedication of MBCT teachers and that there is a long way to go before the principles and practice of MBCT services permeate through to mental health services. Securing greater political and organisational commitment to the provision of interventions such as MBCT that aim to prevent depression and enhance wellbeing remains to be important goals.

It is clear that whilst the implementation of MBCT is in the early stages, in the UK, North America, Scandinavia, Australia and the Benelux countries, there are many examples of people working to overcome implementation barriers. Table 2 (Kuyken et al. 2012) presents summary of the guiding principles for MBCT implementation that are drawn from respondents reporting of both barriers and facilitators to local implementation. The Nutely et al. (2007) framework is used for structuring these recommendations. The framework is based on extensive review of the evidence of what contributes to the likelihood of evidenced-based practice flourishing: research must be translated, ownership is key, enthusiasts are needed, a contextual analysis is essential, credibility must be established and leadership is needed (see Table 2). An MBCT Implementation Resource kit provides detailed material to support development in these areas (Kuyken et al. 2012).

**Table 2:** The Bangor, Exeter and Oxford guiding principles for MBCT implementation (Kuyken et al. 2012)

Guiding principles	How this relates to MBCT
Research needs to be translated	- Make local decisions about target populations/inclusion/exclusion criteria
	- Base decisions on definitive and emerging MBCT evidence for MBCT and on local service priorities
- Research needs to be accessible to services. Typically, this involves tailoring the research and consensus development at a local level	- Consider and map out how new MBCT service will sit alongside existing care pathways

Ownership is critical	- Engage key stakeholders in service planning and commissioning
- Ownership of the research or of the implementation process is likely to positively affect uptake	- Offer taster sessions/intern places for stakeholders to communicate MBCT's aims and intentions
- System based, top-down approaches that “force” research use in organisation can negatively affect uptake	- Support grassroots interest through experiential opportunities to take mindfulness classes
	- Develop local networks for interested clinicians and stakeholders

Enthusiasts are key	- Identify one or more “champion(s)” with adequate knowledge and access to key networks
- People who are enthusiastic about the issue/topic/practice can act as champions and promote new ideas	- Champions are needed both within the organisation and external to the organisation
	- Former participants in MBCT classes can be compelling advocates

Conduct an analysis of context	- Analyse local context to identify implementation barriers and facilitators
- An analysis of the context of implementation prior to designing the strategy can facilitate a particularised approach through the targeting of local barriers and facilitators	- Set up an implementation steering group to systematically address local barriers and facilitators in the range of challenge areas and to develop and oversee the new service until it is fully embedded

Ensure credibility	- Ensure that key evidence and national guidance on MBCT is clearly conveyed to staff by a credible champion
- Research use is enhanced by credible evidence, credible champions/opinion leaders and a commitment to process	- Set up appropriate and realistic service evaluation
	- Ensure evaluation data are routinely collected and reported to key stakeholders

Provide leadership	- An overall MBCT service lead is required who can provide clear leadership
- Strong and facilitative leaders at project and organisational level can lend strategic support and authority to the process	- Leadership is needed on a strategic and a clinical level
	- Strategic leaders within the organisation's management should ideally have experiential understanding of MBCT
	- Clinical leaders need in-depth training in MBCT, so they can teach the course and support other staff in developing their skills through supervision and mentoring
	- Leadership on good practice governance is needed using national guidance and contextualising it locally

Provide adequate support/resources	- Identify appropriate and adequately trained staff to run MBCT classes who at minimum meet the UK good practice recommendations (UK Network 2011)
- Implementation needs adequate resources and support including financial, human (dedicated project leaders) and appropriate equipment	- Using epidemiological data, it is estimated that a population of 200,000 would need 2 full-time MBCT teachers to provide a service (Patten and Meadows 2009). If the service is being offered to a broader client group that is recommended by NICE, then more teachers will be required
	- Support and cultivate competent MBCT teachers
	- Support and cultivate (though the classes and reunions) former MBCT participants
	- Secure staff time to prepare and run classes
	- Secure staff time for screening, assessment and orientation of participants
	- Secure staff time for providing some individual participant support between sessions in person or via phone, text or email
	- Put in place required training, supervision for ongoing development and adherence to good practice standards
	- Ensure that a fit for purpose room is available
	Ensure that an ongoing supply of meditation recordings and participant handouts is available
	- Secure administrative support for setting up classes and preparing class handouts
	- Ensure that equipment for sessions is available

Develop opportunities for integration	- Integrate MBCT implementation strategy with local and national strategies for increasing access to psychological therapies
- Activities, changes and new practices need to be integrated into the organisation's systems and processes to enhance their sustainability. Initiatives that fit with strategic priorities are more likely to be given/allocated adequate resources and support	- Identify appropriate imperatives for MBCT, such as the NICE depression guidance, health economic data or local strategic initiatives
	- Establish a service pathway from referral through to discharge and communicate this effectively to all stakeholders
	- Cultivate relationships with referrers
	- Enhance service sustainability by promoting it and integrate it with other strategic priorities

Based on Nutely's (2007) synthesis of factors that shape evidence use in public services. © Kuyken et al. (2012)

## Conclusions

It is only 10 years since the MBCT manual was published (Segal et al. 2002), 8 years since the first evidence synthesis recommended MBCT National Institute for Health and Clinical Excellence (NICE), 2004, Guideline 23, P. 76 and only 2 years since robust approaches to MBCT teacher training were published (Crane et al. 2010). Implementation is the next challenge for this emerging field, and in the context of the early stage in the development of MBCT, it is not surprising that there are challenges to be overcome. It is however important that these challenges are systematically considered and addressed so that implementation of MBCT reflects best practice and ensures that the promising results obtained in research contexts are replicated in routine clinical practice.

The current inconsistent approach to implementation contributes to health inequalities and misses an opportunity to translate evidence into practice (Ward et al. 2009). The UK provides a potential exemplar because there is a national health service, national guidance in place recommending MBCT's implementation, a large proportion of the evidence of its efficacy was generated in the UK, there are three universities focusing on MBCT training and research and there is a strong grassroots interest.

We hope that the preliminary MBCT implementation resource kit (Kuyken et al. 2012) will support further implementation and will itself be tested, expanded and refined. Gathering outcome data on the effectiveness of MBCT delivered in naturalistic settings and benchmarking, these results against outcomes from randomised controlled trials will further support implementation.

The interest in mindfulness-based interventions is rapidly expanding. The next step in supporting the implementation of mindfulness-based interventions is to expand the research on MBCT to other populations and other settings and to investigate the efficacy, effectiveness and implementation of the closely aligned intervention mindfulness-based stress reduction.
